# Supplementary material for: Low Pt loading for high-performance fuel cell electrodes enabled by hydrogen-bonding microporous polymer binders
Source: Nat Commun. 2022 Dec 8;13:7577. doi: 10.1038/s41467-022-34489-x (PMC9732346; doi:10.1038/s41467-022-34489-x)
Supplement: Supplementary file 1 — Supplementary Information [file 41467_2022_34489_MOESM1_ESM.pdf]

Low Pt Loading for High-performance Fuel Cell Electrodes Enabled by Hydrogen-bonding Microporous Polymer Binders

Hongying Tang,[1,2,3] Kang Geng,[1] David Aili,[3] Qing Ju,[1] Ji Pan,[4] Ge Chao,[1] Xi Yin,[1] Xiang Guo,[1] Qingfeng Li,[3,***] Nanwen Li[1,*]**

[1] State Key Laboratory of Coal Conversion, Institute of Coal Chemistry, Chinese Academy of Sciences, Taiyuan, China.

[2] Tianjin Key Laboratory of Water Resources and Environment, Tianjin Normal University, Tianjin, China.

[3] Department of Energy Conversion and Storage, Technical University of Denmark, Elektrovej, Building 375, 2800 Lyngby, Denmark.

[4] College of Chemistry, Chemical Engineering and Materials Science, Soochow University, No. 199 Renai Road, Suzhou, China.

*Correspondence: linanwen@sxicc.ac.cn.

**Correspondence: hytang@tjnu.edu.cn

***Correspondence: qfli@dtu.dk.

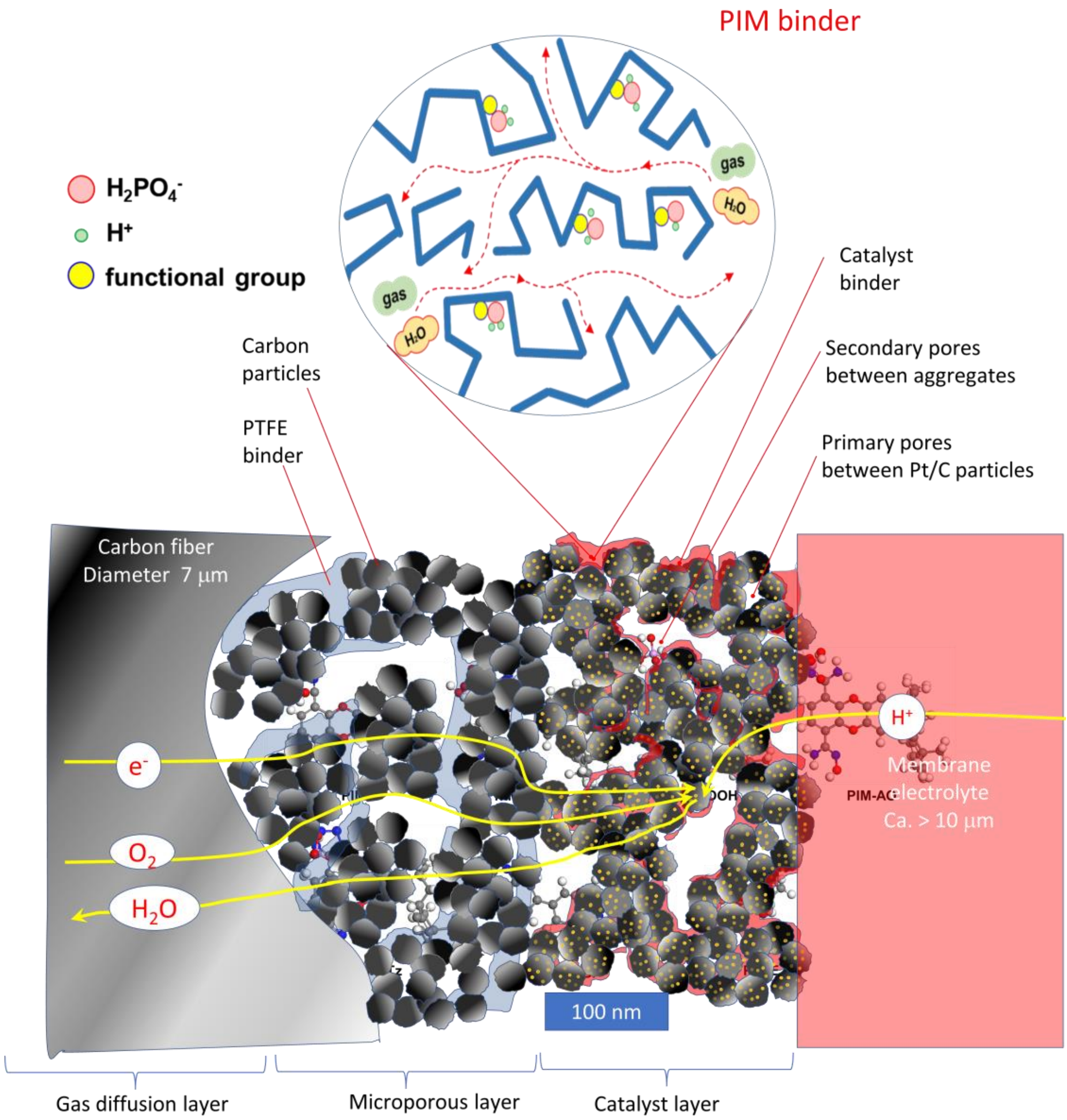

Supplementary Figure 1. Schematic representation of the three-phase boundary of the catalyst layer in a fuel cell MEA and the characteristic feature of the PIM catalyst binder which possesses intrinsic microporosity and acidophilic sites for phosphoric acid binding.

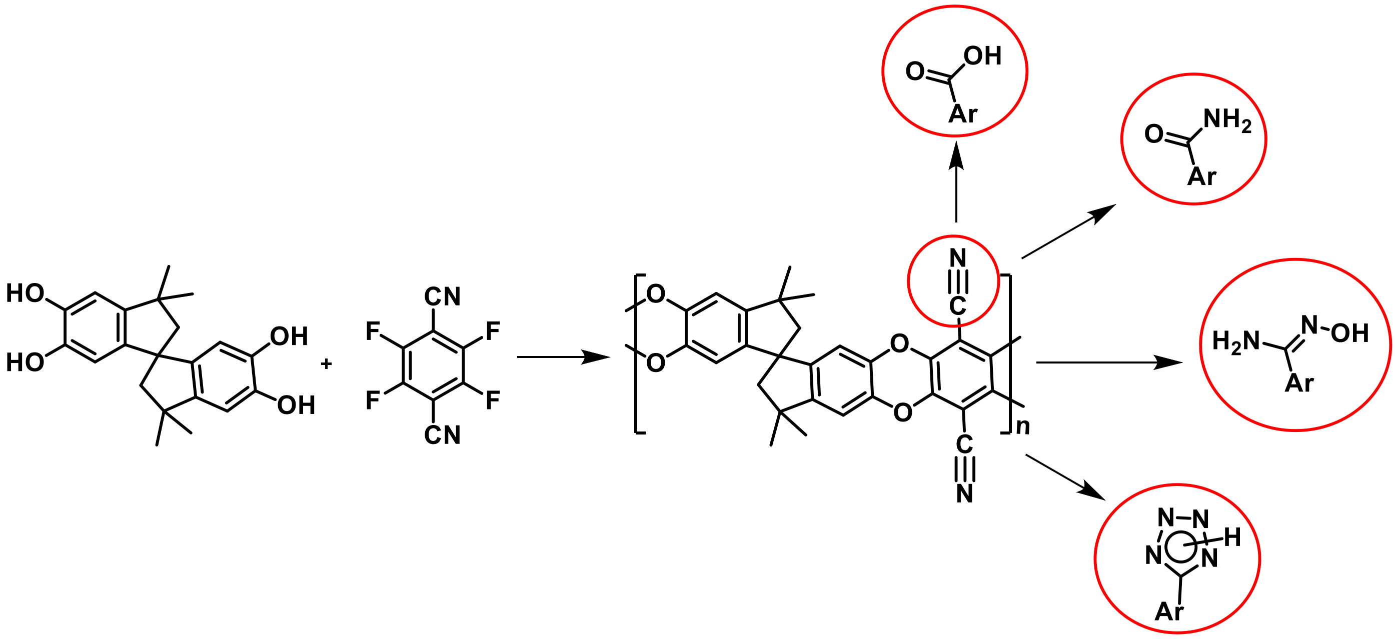

Supplementary Figure 2. Synthesis and structures of PIM-1 and its derivatives

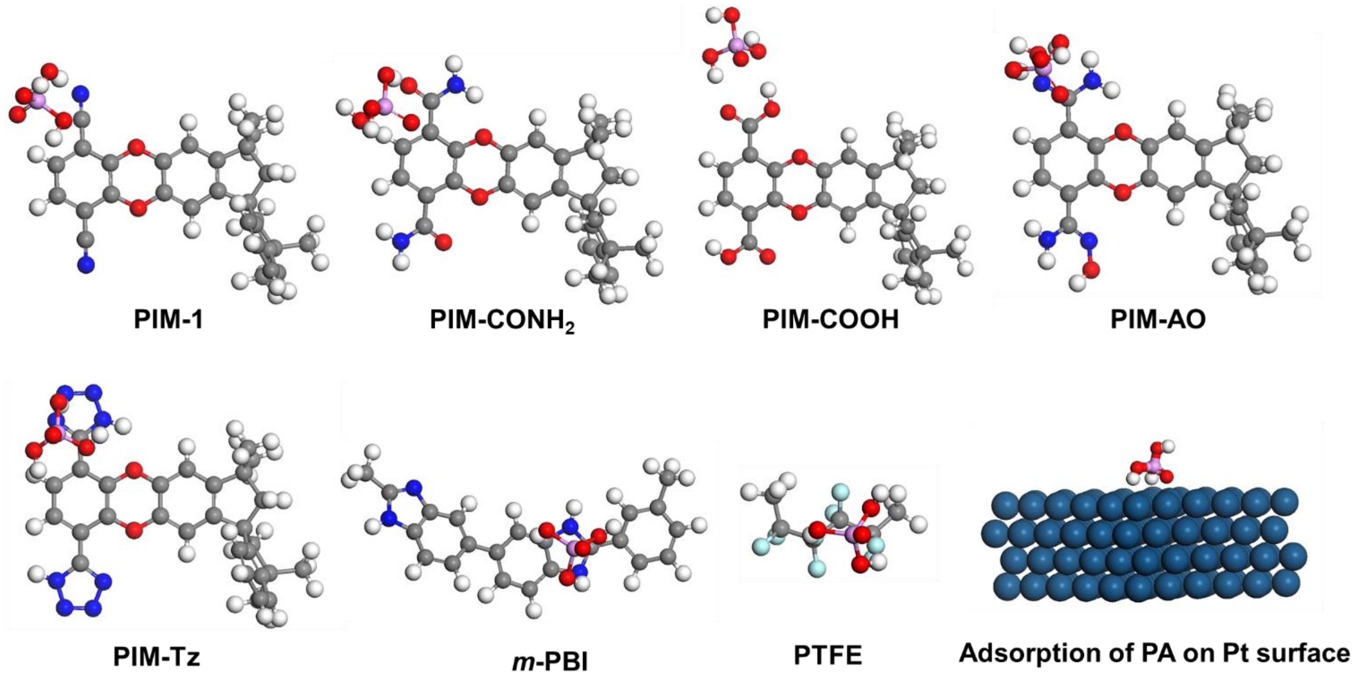

Supplementary Figure 3. Optimized structures of PIMs for calculation of binding energy with phosphoric acid.

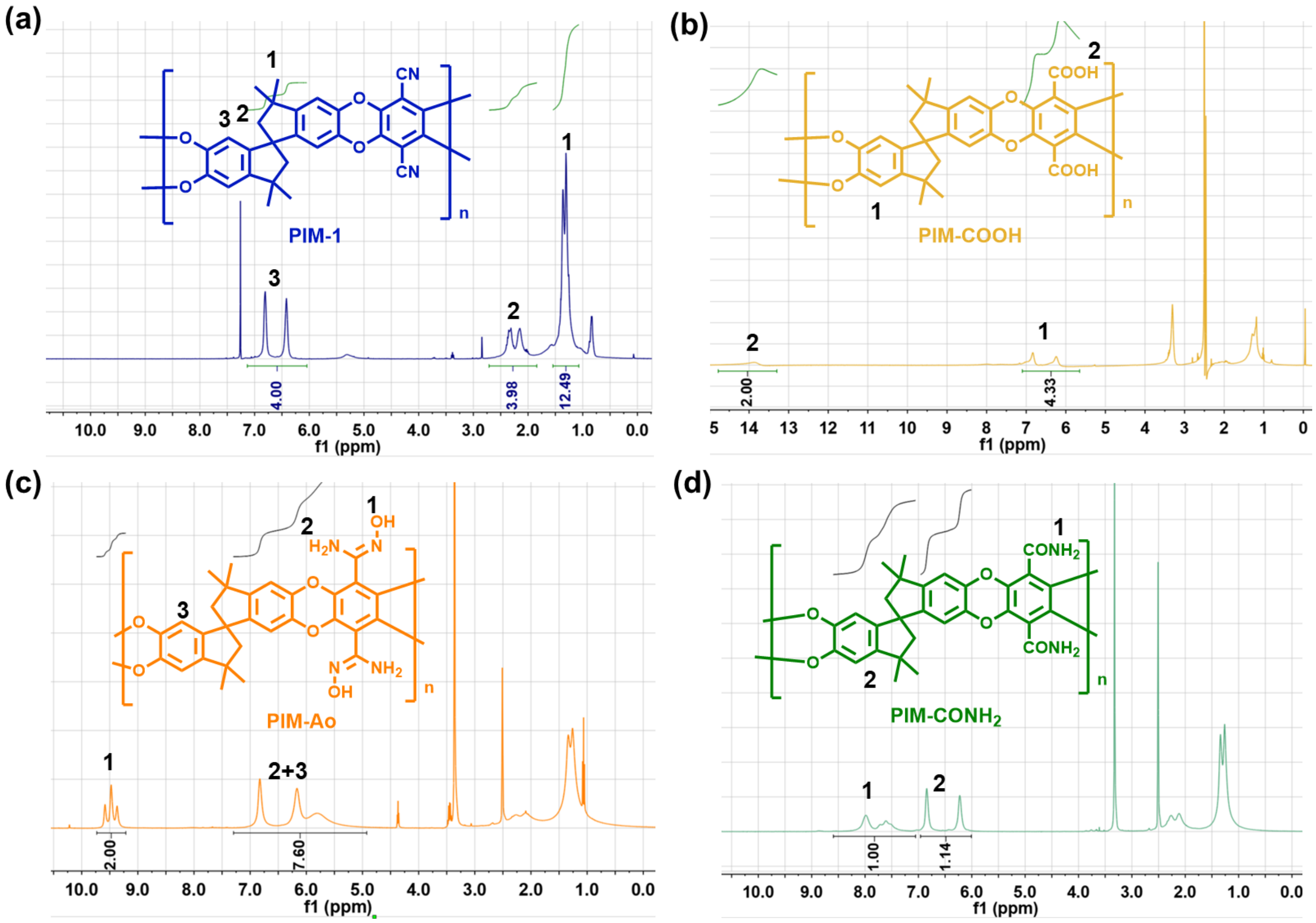

Supplementary Figure 4. ^{1}H NMR spectra of the PIMs.

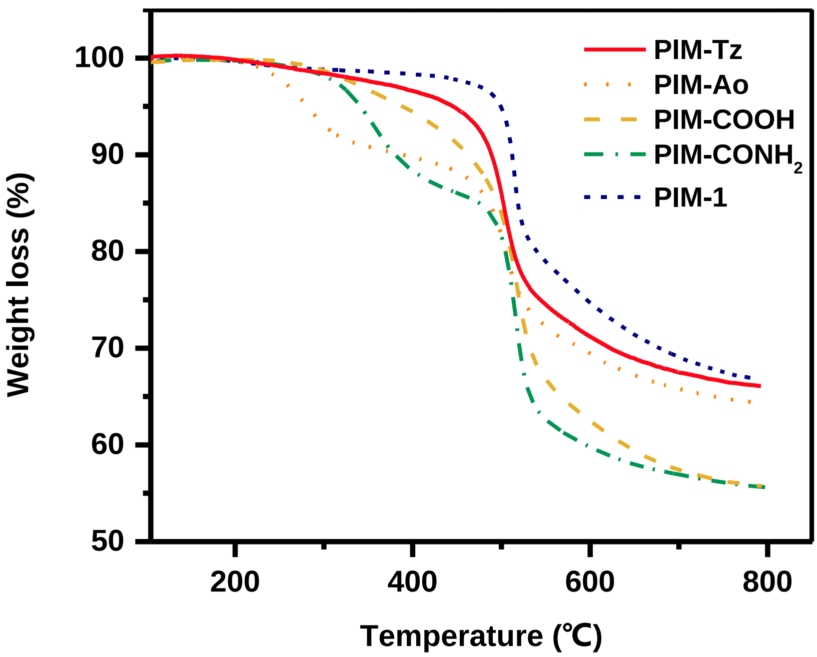

Supplemetary Figure 5. Thermogravimetric analysis of PIMs.

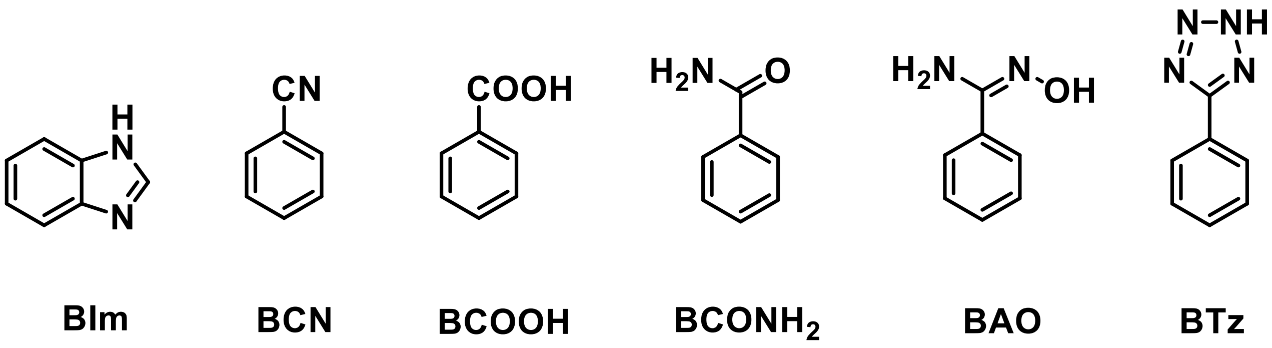

Supplementary Figure 6. Structures of the model monomers.

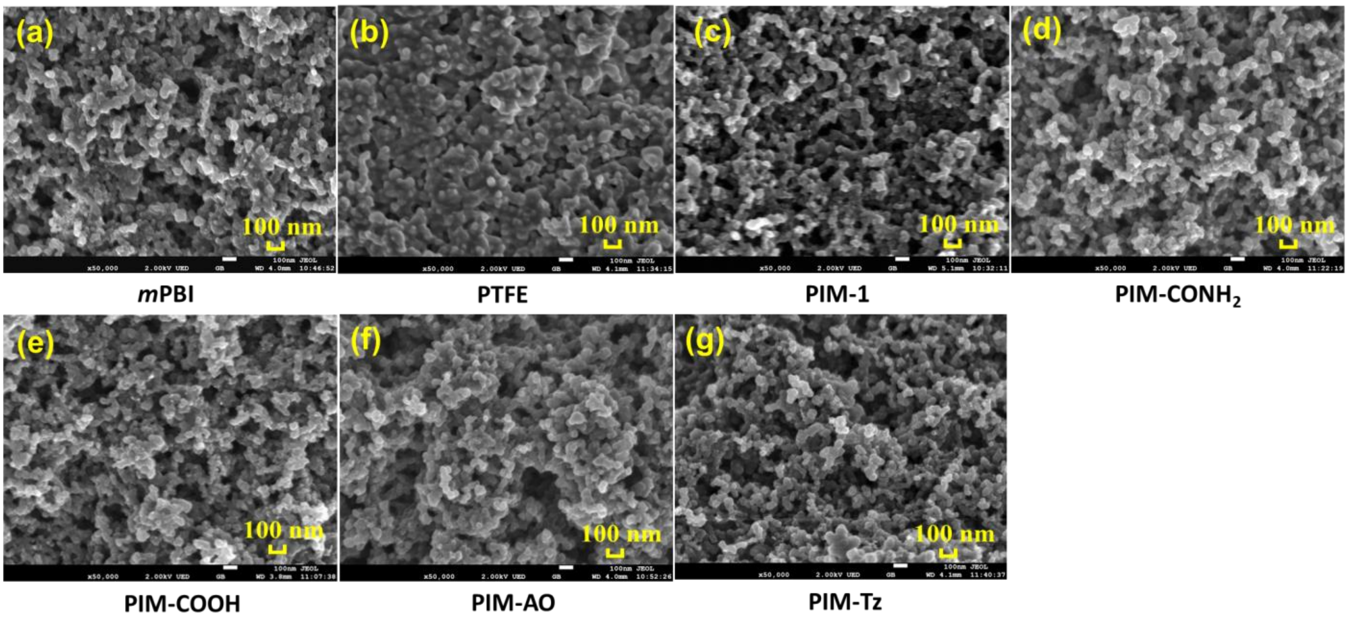

Supplementary Figure 7. Cross sectional images of catalyst layers with different binder materials, and the present SEM of GDEs using PTFE and *m*PBI as binders for comparison. The content of the binder materials is 20 wt% of total solid in the catalyst ink.

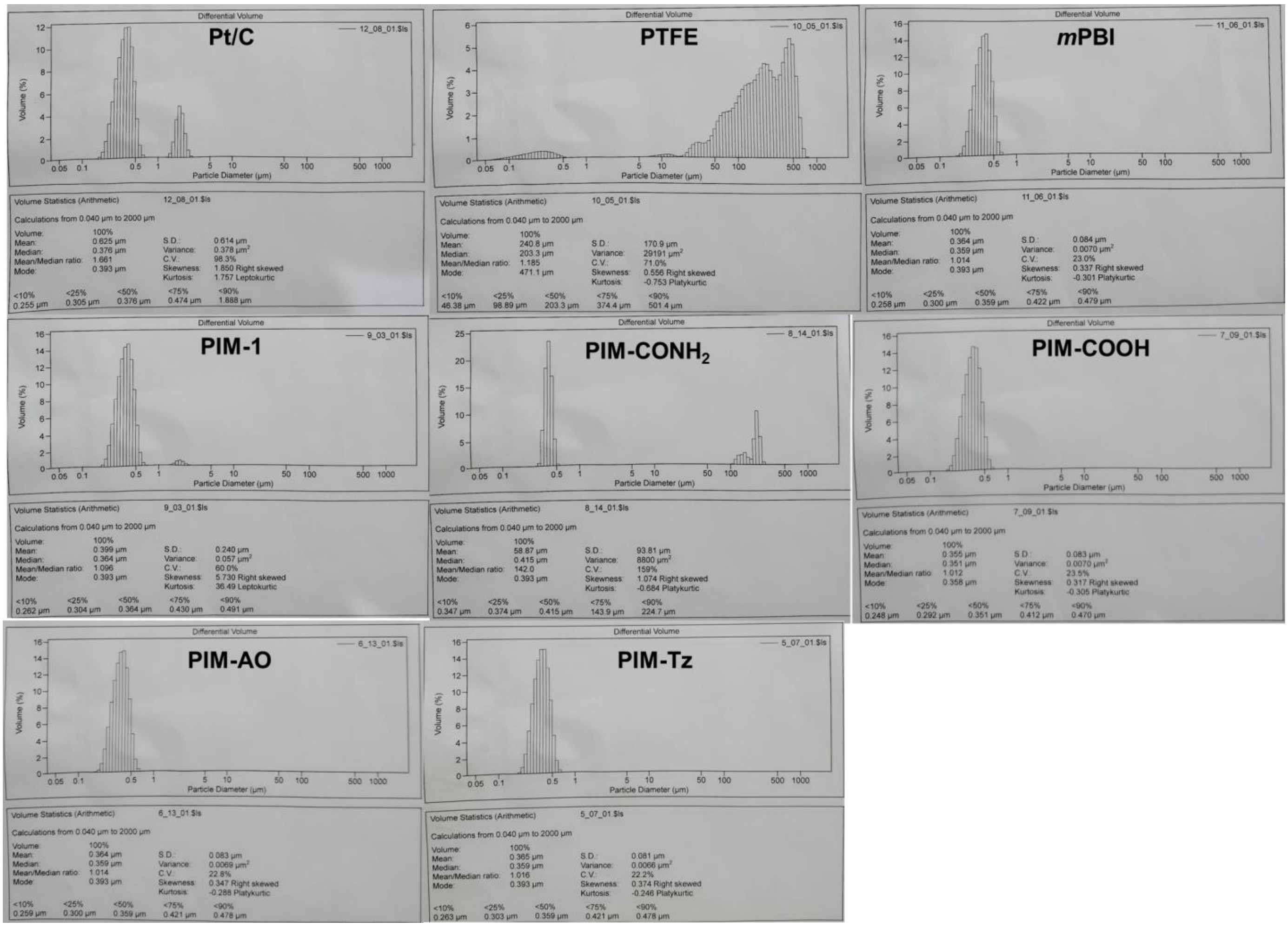

Supplementary Figure 8. Laser scattering particle size analyzer data.

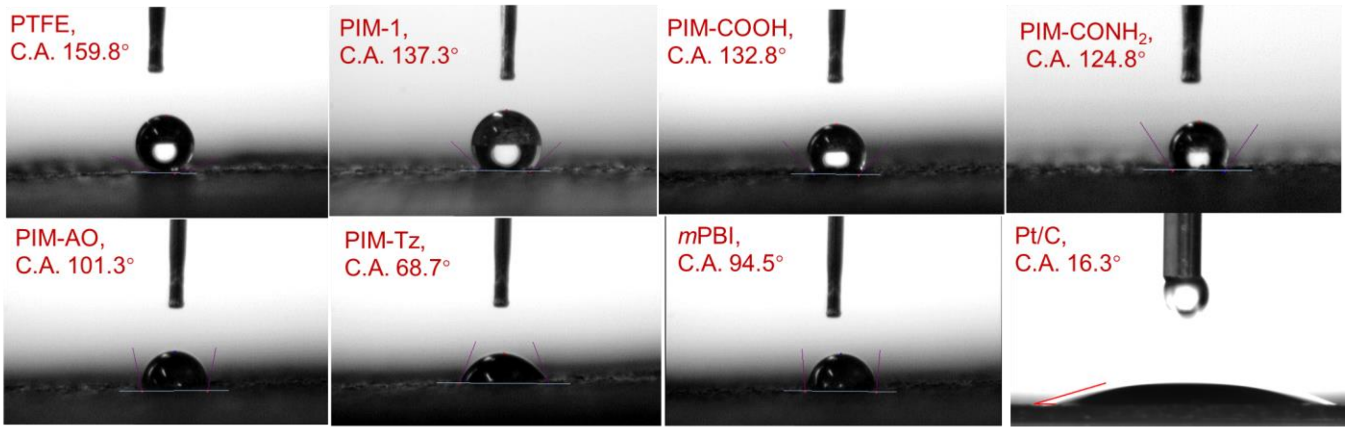

Supplementary Figure 9. The water contact angle of the GDEs with different binders. The Pt GDE means there is only Pt/C in the catalyst layer. The content of the binder in the catalyst layer is 20 wt% of total solid in the catalyst ink.

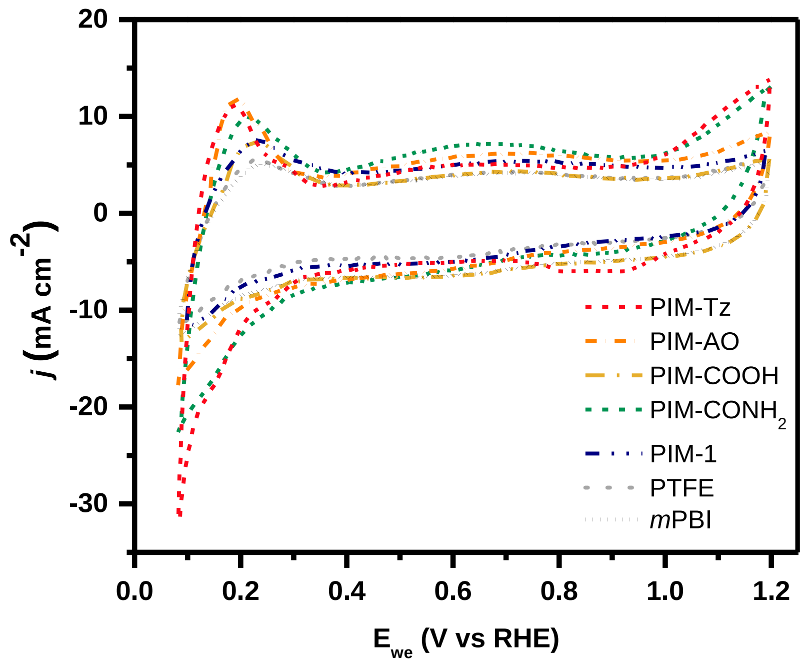

Supplementary Figure 10. Cyclic voltammograms of the MEAs with different GDEs, anode 30 sccm dry H_2 flow, cathode 200 sccm dry N_2 flow at ambient pressure. The test conditions are listed in Supplementary Table 1.

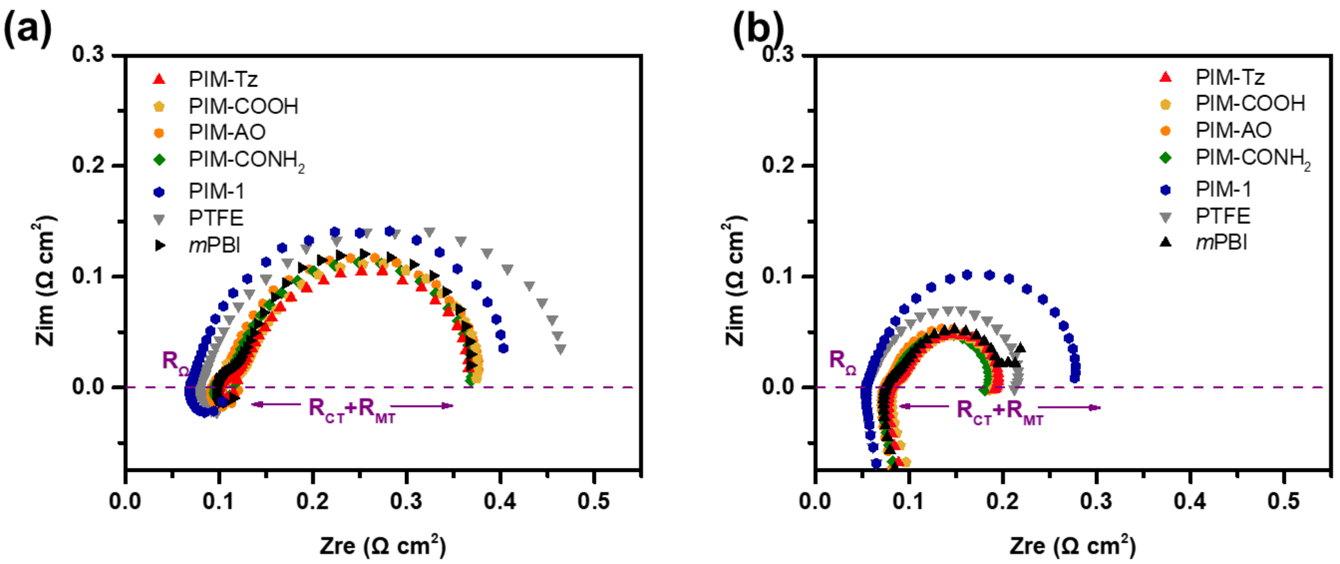

Supplementary Figure 11. In situ impedance curves of the MEAs with different binder materials at (a) 0.3 A cm^{-2} and (b) 1.0 A cm^{-1} with frequency ranging from 1 Hz to 10 KHz. Test conditions are listed in Supplementary 1.

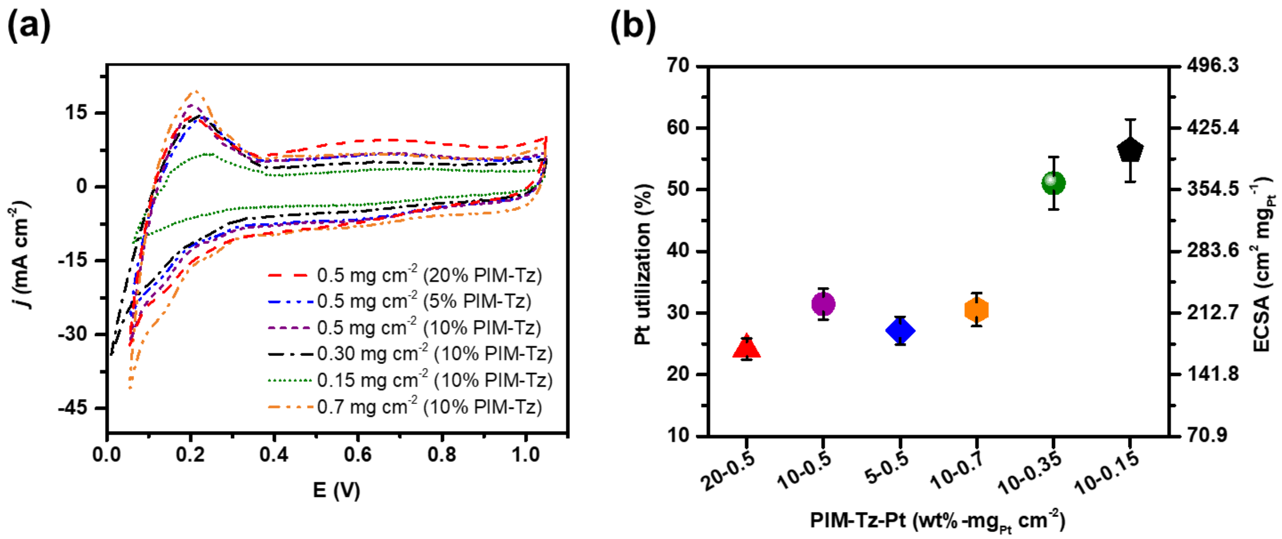

Supplementary Figure 12. (a) Cyclic voltammograms with 30 sccm dry H_2 flow to the anode and and 200 sccm dry N_2 flow to the cathode at ambient pressure, and (b) Pt utilization of PIM-Tz MEAs with different catalyst and binder contents. The error bars in (b) indicate the integral of the hydrogen desorption area of the CV curves. The test conditions are listed in Supplementary Table 1.

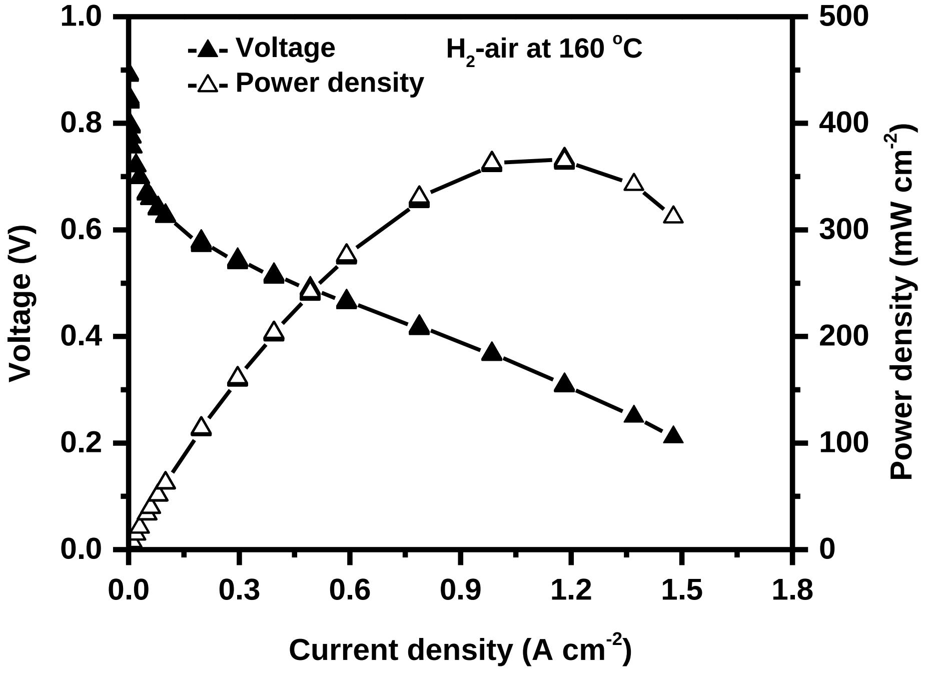

Supplementary Figure 13. Cell performance of the MEA with PIM-Tz binder material of 10% and catalyst loading of 0.15 mg_{Pt} cm^{-2} at 160 °C. The test conditions are listed in Supplementary Table 1.

Supplementary Table 1, The test conditions of the single cells at 160 °C with no extra humidification and no back pressure.

Binder	Membrane	Catalyst loading (mg_{Pt} cm^{-2})[a]	Binder content (wt%)[b]	Flow rate of H_2-O_2 (sccm)
**The cell test conditions using different binder materials in the catalyst layer**				
**PIM-Tz**	mPBI/PA (78 μm)	0.500	20	30c
**PIM-COOH**	*m*PBI/PA (80 μm)	0.501	20	30
**PIM-AO**	*m*PBI/PA (79 μm)	0.505	20	30
**PIM-$CONH_2$**	*m*PBI/PA (81 μm)	0.500	20	30
**PIM-1**	*m*PBI/PA (81 μm)	0.488	20	30
**PTFE**	*m*PBI/PA (80 μm)	0.506	20	30
**mPBI**	*m*PBI/PA (77 μm)	0.510	20	30
**The cell test conditions using PIM-Tz binder in the catalyst layer**				
**PIM-Tz**	*m*PBI/PA (78 μm)	0.501	5	30
**PIM-Tz**	*m*PBI/PA (79 μm)	0.499	10	30
**PIM-Tz**	*m*PBI/PA (80 μm)	0.500	20	30
**PIM-Tz**	*m*PBI/PA (78 μm)	0.700	10	30
**PIM-Tz**	*m*PBI/PA (77 μm)	0.350	10	30
**PIM-Tz**	*m*PBI/PA (78 μm)	0.150	10	30
**The cell test conditions using PIM-Tz binder in the catalyst layer with H_2-air fuel**				
**PIM-Tz**	*m*PBI/PA (78 μm)	0.350	10	30
**PIM-Tz**	*m*PBI/PA (77 μm)	0.150	10	30

[a] Catalyst loading in both cathode and anode. [b] Binder content refers to the binder as a percentage of the solid in the catalyst slurry.

Supplementary Table 2. Cell performance analysis, overpotential values obtained at 0.3 and 1.0 A cm^{-2}.

Binder	OCV (mV)	0.3A/cm^2			1.0 A cm^{-2}				j @ 0.90 V (μA cm^{-2} Pt)	Tafel slope (mVdec^{-1})
		E (mV)	η_{act} (mV)	HFR (mΩ cm^2)	E (mV)	HFR (mΩ cm^2)	η_{act} (mV)	$\eta_{mass+CT}$ (mV)		
**PIM-Tz (75%)**	945.6	640.5	470.1	100.0	495.0	80.0	523.9	40.1	7.9	76.8
**PIM-AO**	935.2	625.6	485.2	92.0	465.5	72.5	539.4	55.4	5.1	77.5
**PIM-COOH**	941.0	615.8	498.2	104.6	478.0	83.5	545.6	23.5	5.1	80.6
**PIM-CONH$_2$**	948.6	620.0	495.7	95.9	475.0	75.8	563.7	38.1	4.8	77.7
**PIM-1**	917.4	598.7	521.1	68.9	407.1	54.3	583.3	94.4	4.4	88.9
**PTFE**	940.5	567.1	586.1	79.4	400.0	55.0	651.1	32.9	1.6	92.8
***m*PBI**	928.0	606.4	499.2	97.5	399.0	74.5	548.8	116.7	4.5	70.8

Supplementary Table 3. The cell performance comparison of this work with the data collected from the literature (reactants: H_2-O_2).

Membrane	Binder	Pt loading (mg_{Pt} cm^{-2}) Anode/cathode	PPD[a]@ temperature (mW cm^{-2}@°C)	Pt-mass specific PPD[a,b] (W mg_{Pt}^{-1})	Durability/Degradation rate	Ref.
m-PBI/PA	PIM-Tz	0.5/0.5	828 (160)	1.7	>800 h; (H_2-O_2 cell, 0.3 A cm^{-2}, Anode/Cathode 0.5 mg_{Pt} cm^{-2}, binder content: 20%)	This work
m-PBI/PA	PIM-Tz	0.35/0.35	832 (160)	2.4	600 h; stable (H_2-air cell, 0.3 A cm^{-2}, binder content: 10%)	This work
m-PBI/PA	PIM-Tz	0.15/0.15	570 (160)	3.8	--	This work
***m*-PBI**	PBI or PTFE	N.A.	1000 (200)	1.0 (3 atm)	>2000 h (180 °C); >5000 h, <5 μV h^{-1} (150 °C)	1
***p*-PBI sol-gel membranes**	E-Tek GDE	>1.0/1.0	750 (160)	0.7	4.9–6.3μV h^{-1} (0.2 A cm^{-2})<br>>1000h; 43μV h^{-1} (0.4 A cm^{-2})	2,3
**Celtec®-P 1000**	BASF GDE	0.75-1.0/0.75-1.0	160	N.A.	>6000h; 5–6 μV h^{-1}	4,5
**AB-PBI**	PTFE	1.2/1.2	300 (160)	0.3	6000 h; 25μV h^{-1} (H_2-air)	6,7
**20 W stack (PBI)**	PTFE/PBI	1.0/1.0	300 (150)	0.3	1200 h; no visible degradation (H_2-air)	8
**Poly (aryl sulfone benzimidazole)**	HCOOH, PA and *m*PBI	0.7/0.7	346 (180)	0.5	>2400 h; 2.4–6.4μV h^{-1} (0.3 A cm^{-2})	9

**PBI**	HCOOH, PA and *m*PBI	0.6/0.6	430 (160)	0.7	>1500 h; 1.5 μV h^{-1} (0.3 A cm^{-2})	10
**Thermoset *m*PBI**	*m*PBI	0.58/0.29	350 (160)	1.1	>2000 h; 5–6 μV h^{-1} (0.2 A cm^{-2}); 43 μV h^{-1} (0.6 A cm^{-2})	11
**So-gel PBI (BASF)**	BSF GDE	0.7/1.0	350 (180)	0.4	>6000 h, 4.9–6.3 μV h^{-1} (0.2 A cm^{-2}, 160 °C); 19 μV h^{-1} (180 °C)	12
**PBI & PBI-O-PhT + Zr $(Acac)_4$ composite**	BASF GDE	1.0/1.0	300 (160)	0.3	>2000 h, no visible degradation (0.4 A cm^{-2})	13
**OPBI**	Nafion	1.5/1.5	350 (160)	0.2	780 h; ~25 μV h^{-1} (0.2 A cm^{-2})	14
**Celtec P1000 MEAs**	BASF GDE	>1.0/1.0	N.A. (160)	N.A.	658 h; 200–520 μV h^{-1} (0.4 A cm^{-2}, reformate-air)<br>1105 h; 41–149 μV h^{-1} (single cell)	15
**ABPBI**	PTFE	1.0/1.0	400 (160)	0.4	1000 h; 14 μV h^{-1} (0.2 A cm^{-2}, spiral flow)	16
**PyPBI**	BASF GDE	1.0/1.0	N.A. (180)	N.A.	2300 h; 5.2 μV h^{-1} (0.2 A cm^{-2})	17
**PBI**	PVDF	0.8/0.8	N.A. (150)	N.A.	17,860 h; <4 μV h^{-1} (0.2 A cm^{-2})	18
**Cross-linked AB-PBI**	PVDF	0.8/0.8	N.A. (150)	N.A.	17,500 h; <2 μV h^{-1} (0.2 A cm^{-2})	19
**12 cell (320 cm^{2}) stack**	BASF GDE	>1.0/1.0	N.A. (160-180)	N.A.	3400 h; 24 μV h^{-1} (0.2 A cm^{-2}, 33 % H_2	20

**(BASF MEAs)**					+ 1 % CO)	
**PBI MEA (BASF)**	BASF GDE	>1.0/1.0	N.A. (180)	N.A.	4000 h; 19 μV h^{-1} (0.2 A cm^{-2}); 157 start-up/shutdowns, 480 μV/cycle	21
**Cross Linked-6FPBI**	HCOOH, PA and *m*PBI	0.6/0.6	360 (160)	0.6	2600 h; 63 μV h^{-1} (0.3 A cm^{-2})	22
**Sulfonated PBI**	BASF GDE	1.0/1.0	600 (180)	0.6	~3000 h; 30 μV h^{-1} (0.2 A cm^{-2}, 160 °C)	23
***m/p* PBI copolymer**	>BASF GDE	1.0/1.0	600 (180)	0.6	2 years, ~0.67 μV/h (0.2 A cm^{-2}, 160 °C, H_2-air)	24
**Polysulfonated PBI**	N.A.	0.5/0.5	318 (150 °C, 1 bar)	0.6	190 h (0.2 A cm^{-2}, H_2-air)	25
**Polysulfonated Fluoro-oxyPBI Membranes**	N.A.	0.5/0.5	360 (150 °C, 1 bar)	0.7	800 h (no degradation); 1000 h 14 μV h^{-1} (0.2 A cm^{-2}, H_2-air)	26
**Phosphonated-PPFS/PBI composites**	Johnson Matthey GDE	0.4/0.4	780 (70 °C, 100% RH)	1.95	70 h (0.5 V, 70 °C)	27
**Tz-poly(arylene ether)**	PTFE	1.03/1.03	287 (160)	0.3	325 h (0.6 A cm^{-2}, H_2-air)	28
**Quaternary ammonium-biphosphate ion pairs**	QASOH	0.6/0.4 Pt-Ru	800 (180)	2.0	200 h (160 °C, H_2-air); 0.33 mA cm^2 h^{-1} (120 °C, P_{H_2O} = 3.6 kPa)	29,30
**Quaternary ammonium-biphosphate ion pairs**	Phosphonated PPSF	0.5/0.6	1.74 (240 °C, 147.1 kPa)	2.9	>550 h; 0.35 μV h^{-1} (0.6 A cm^{-2}, 160 °C, H_2-air, 147.1 kPa)	31

**PA/PBI**			300-700 (120-200 °C, 147.1 Kpa)	0.5-1.2		
**Polycation−Polybenzimidazole Blends**	QPPSf	0.5/0.5	680 （220）	1.4	30 h (220 °C), >84 h (180 °C), (0.2 A cm^{-2})	32
**Poly(arylene piperidine)s**	PTFE	1.2/1.2	1220 (180 °C, 0.15 MPa)	1.0	1500 h (0.12 A cm^{-2}, 150 °C H_2-air)	33
**PBI/SiO_2**	BSF	1.0/1.0	283 (230)	0.3	100 h (0.6 V, 240 °C, H_2 containing 6.3% CO)	34
**Self-crosslinked PEI-PSF**	N.A.	N.A.	200 (150)	N.A.	18-80 h (0.15 A cm^{-2})	35
**PSF-QA**	PTFE	0.55/0.55	>400 (160)	>0.7	<30 h (0.2 A cm^{-2})	36
***m*PBI**	Phosphonated PPSU	0.5/0.5	242 (160)	0.5	200 h, no significant degradation (0.2 A cm^{-2})	37
**F_6PBI**	N.A.	1.0/1.0	420 (160)	0.4	--	38,39
**PFCB–PBI**	N.A.	1.0/1.0	210 (140)	0.2	--	40
**2HOPBI**	BASF GDE	1.0/1.0	130 (160)	0.1	--	41
**F_6PBI, SO_2PBI and blends**	HCOOH, PA and *m*PBI	0.6/0.6	450 (200)	0.9	--	42
**Crosslinked NH_2PBI**	N.A.	N.A.	120 (160)	N.A.	--	43,44
**Hyperbranched PBI**	N.A.	N.A.	60 (160)	N.A.	--	45,46

**Asymmetric 6FPBI**	PBI	1.0/1.0	280 (170)	0.3	--	47
**Branched *p*PBI-b-F6-PBI**	N.A.	N.A.	713 (160)	N.A.	--	48
**Bipy-PBI**	N.A.	N.A.	779 (120)	N.A.	--	49
**PIM-1 reinforced PBI alloy membranes**	HCOOH, PA and OPBI	0.6/0.6	438 (160)	0.7	--	50
**Imidazole-riched Crosslinked Networks**	N.A.	0.6/0.6	533 (160)	0.9	--	51
**SiO_2/imidazole-cPBI**	PA/ PBI	0.6/0.6	497 (160)	0.8	--	52
**Arylether-type benzimidazolyl-OPBI**	N.A.	0.6/0.6	443 (160)	0.7	--	53
**Ether linked bulky pendant- OPBI**	N.A.	N.A.	320 (160 °C, H_2/air)	N.A.	--	54
**Cross-linked triazole-XTPPO**	N.A.	N.A.	220 (150)	N.A.	--	55
**PTFE/Quaternized PVBzCl$^-$**	qPVB/Cl$^-$ and PVDF	0.5/0.5	360 (175)	0.7	--	56
**PES-PVP blends**	PVP	1.1/0.5	850 (180)	1.7	--	57,58
**PVDF-PVP blends**	PVP	0.5/0.5	530 (180)	1.1	--	59
**PBI/PA**	PBI/ZnO	0.5/0.5	∽600 (200)	∽1.2	--	60

**TB/PA**	PTFE	0.5/0.5	815 (-20-200)	1.6	--	61
**Quaternary ammonium-biphosphate ion pairs**	Nafion–PWN	0.5 (PtRu/C) /0.7 (Pt/C)	500-2000 (80-200 °C, 170 kPa)	0.7-3.0	>2500 h (0.6 A cm^{-2}, 160 °C, H_2-air, 148 kPa)	62

[a] **Peak power density;**

[b] **Pt-mass specific PPD is calculated according to the Eq.:** $Pt - mass\ specific\ power\ density\ (W\ mg_{Pt}^{-1}) = \frac{Peak\ Power\ density\ (W\ cm^{-2})}{Pt\ loading\ of\ the\ cathode\ (mg_{Pt} cm^{-2})}$

Supplementary Table 4. Comparison of MEAs performance collected from the literature and this work for cathode Pt loadings ⩽ 0.35 mgPt cm^{-2} (Reactants: H_2/Air@160 °C)

Binder	Membrane	CL deposition method/electrode type	Pt loading (mg cm^{-2}) (anode/cathode)	PPD[a] (W cm^{-2})	Current density @ 0.6 V (A cm^{-2})	Pt-mass specific PPD[a,b] (W mg_{Pt}^{-1})	Ref.
**PIM-Tz**	***m*PBI**	**Spray/CCS**	**0.35/0.35**	**0.535**	**~0.34**	**1.5**	**This work**
**PIM-Tz**	***m*PBI**	**Spray/CCS**	**0.15/0.15**	**0.360**	**~0.15**	**2.4**	**This work**
**DPS GDE**	*m*PBI	Spray/CCS	**0.9/0.9**	**0.421**	**~0.4**	**0.5**	**This work**
**PVDF**	ABPBI	Ultrasonic spraying/CCM	0.3/0.5	~0.277	~ 0.1	~0.6	63
**PVDF**	ABPBI	Ultrasonic spraying/CCM	0.5/0.3	~0.228	~ 0.1	~0.8	64
**PTFE**	ABPBI	Ultrasonic spraying/CCS	0.7/0.3	0.425	~ 0.2	1.4	65
**PTFE**	PBI	Electrospraying/CCS	0.25/0.25	0.085	~ 0.04	0.3	66
**PTFE**	ABPBI	Doctor blade/CCS	0.95/0.16	0.270	~ 0.17	1.7	67
**PVDF**	ABPBI	Ultrasonic spraying/CCS	0.138/0.138	0.216	~ 0.05	1.6	68
**binderless**	PBI	Ultrasonic spraying/CCS	0.48/0.11	0.258	0.06	2.3	69
**N.A.**	PBI	Ultrasonic spraying/CCS	0.1/0.1	0.321	~ 0.1	3.2	70
**PTFE**	PBI	Ultrasonic spraying/CCS	0.1/0.05	0.350 (180 °C)	~ 0.14	7	71

**PTFE**	PBI	Electrospraying/CCS	0.1/0.1	0.420 (180 °C)	0.2	4.2	72
**PTFE**	PBI	Reactive spray deposition/CCS	0.05/0.05	~ 0.230	~ 0.16	~ 4.6	73

[a] **Peak power density;**

[b] **Pt-mass specific PPD is calculated according to the Eq.:** $\boldsymbol{Pt - mass\ specific\ power\ density\ (W\ mg_{Pt}{}^{-1}) = \frac{Peak\ Power\ density\ (W\ cm^{-2})}{Pt\ loading\ of\ the\ cathode\ (mg_{Pt}cm^{-2})}}$

References:

1 Li, Q. *et al.* PBI-based polymer membranes for high temperature fuel cells preparation, characterizations, and fuel cell demonstrations. *Fuel Cells,* **4**, 147-159, doi:10.1002/fuce.200400020 (2004).

2 Yu, S. *et al*. Durability studies of PBI-based high temperature PEMFCs. *Fuel Cells,* **8**, 165-174, doi:10.1002/fuce.200800024 (2008).

3 Xiao, L. *et al.* High-temperature polybenzimidazole fuel cell membranes via a sol-gel process. *Chem. Mater.,* **17**, 5328–5333 (2005).

4 Schmidt, T. *et al.* Durability and reliability in high-temperature reformed hydrogen PEFCs. *ECS Trans.,* **3**, 861-869, doi:10.1149/1.2356204 (2006).

5 Schmidt, T. *et al.* Properties of high-temperature PEFC Celtec (R)-P 1000 MEAs in start/stop operation mode. *J. Power Sources,* **176**, 428-434, doi:10.1016/j.jpowsour.2007.08.0 (2008).

6 Wannek, C. *et al.* Durability of ABPBI-based MEAs for high temperature PEMFCs at different operating conditions. *Fuel Cells,* **8**, doi:10.1002/fuce.200700059 (2008).

7 Stolten, D. *et al.* Strategy, status, and outlook for HTPEFC development for APU application (Abstract 162). *Fuel Cell Seminar, 2007,* **San Antonio**, 15-19 (2007).

8 Lee, H.-J. *et al.* Demonstration of a 20 W class high-temperature polymer electrolyte fuel cell stack with novel fabrication of a membrane electrode assembly. *Int. J. Hydrog. Energy,* **36**, 5521-5526, doi:10.1016/j.ijhydene.2011.02.014 (2011).

9 Yang, J. *et al.* Synthesis and properties of poly(aryl sulfone benzimidazole) and its copolymers for high temperature membrane electrolytes for fuel cells. *J. Mater. Chem.,* **22**, 11185, doi:10.1039/c2jm30217a (2012).

10 JS, Y. *et al.* High molecular weight polybenzimidazole membranes for high temperature PEMFC. *Fuel Cells,* **14**, 7-15, doi:10.1002/fuce.201300070 (2014).

11 Aili, D. *et al.* Thermal curing of PBI membranes for high temperature PEM fuel cells. *J. Mater. Chem.,* **22**, 5444–5453, doi:10.1039/c2jm14774b (2012).

12 Galbiati, S. *et al.* Degradation in phosphoric acid doped polymer fuel cells: A 6000 h parametric investigation. *Int. J. Hydrog. Energy,* **38**, 6469-6480, doi:10.1016/j.ijhydene.2013.03.012 (2013).

13 Kondratenko, M. S. *et al.* Novel composite Zr/PBI-O-PhT membranes for HT-PEFC applications. *Beilstein J. Nanotechnol.* **4**, 481-492, doi:10.3762/bjnano.4.57 (2013).

14 Modestov, A. D. *et al.* Degradation of high temperature MEA with PBI-H_3PO_4 membrane in a life test. *Electrochim. Acta,* **54**, 7121-7127, doi:10.1016/j.electacta.2009.07.031 (2009).

15 Moçotéguy, P. *et al.* Long term testing in continuous mode of HT-PEMFC based H_3PO_4/PBI Celtec-P MEAs for μ-CHP applications. *Fuel Cells,* **9**, 325-348, doi:10.1002/fuce.200800134 (2009).

16 Liu, F. *et al.* Effect of spiral flow field design on performance and durability of HT-PEFCs. *J. Electrochem. Soc.,* **160**, F892-F897, doi:10.1149/2.116308jes (2013).

17 Molleo, M. *et al.* High polymer content 3,5-pyridine-polybenzimidazole copolymer membranes with improved compressive properties. *Fuel Cells,* **14**, 16-25, doi:10.1002/fuce.201300202 (2014).

18 Oono, Y. *et al.* Long-term cell degradation mechanism in high-temperature proton exchange membrane fuel cells. *J. Power Sources,* **210**, 366-373, doi:10.1016/j.jpowsour.2012.02.098 (2012).

19 Oono, Y. *et al.* Prolongation of lifetime of high temperature proton exchange membrane fuel cells. *J. Power Sources,* **241**, 87-93, doi:10.1016/j.jpowsour.2013.03.122 (2013).

20 Janßen, H. *et al.* Development of HT-PEFC stacks in the kW range. *Int. J. Hydrog. Energy,* **38**, 4705-4713, doi:10.1016/j.ijhydene.2013.01.127 (2013).

21 Hartnig, C. *et al.* Simulated start–stop as a rapid aging tool for polymer electrolyte fuel cell electrodes. *J. Power Sources,* **196**, 5564-5572, doi:10.1016/j.jpowsour.2011.01.044 (2011).

22 Yang, Y. *et al.* Crosslinked hexafluoropropylidene polybenzimidazole membranes with chloromethyl polysulfone for fuel cell applications. *Adv. Energy Mater.,* **3**, 622-630, doi:10.1002/aenm.201200710 (2013).

23 Mader, J. A. *et al.* Sulfonated polybenzimidazoles for high temperature PEM fuel cells. *Macromolecules,* **43**, 6706-

6715, doi:10.1021/ma1009098 (2010).

24 Pingitore, A. T. *et al.* Durable high polymer content m/p-polybenzimidazole membranes for extended lifetime electrochemical devices. *ACS Appl. Energy Mater.,* **2**, 1720-1726, doi:10.1021/acsaem.8b01820 (2019).

25 Angioni, S. *et al.* Polysulfonation of PBI-based membranes for HT-PEMFCs: a possible way to maintain high proton transport at a low H_3PO_4 doping level. *J. Mater. Chem. A,* **2**, 663-671, doi:10.1039/c3ta12200j (2014).

26 Villa, D. C. *et al.* Polysulfonated fluoro-oxyPBI membranes for PEMFCs: an efficient strategy to achieve good fuel cell performances with Low H_3PO_4 doping levels. *Adv. Energy Mater.,* **4**, 1301949, doi:10.1002/aenm.201301949 (2014).

27 Atanasov, V. *et al.* Phosphonic acid functionalized poly(pentafluorostyrene) as polyelectrolyte membrane for fuel cell application. *J. Power Sources,* **343**, 364-372, doi:10.1016/j.jpowsour.2017.01.085 (2017).

28 Henkensmeier, D. *et al.* Tetrazole substituted polymers for high temperature polymer electrolyte fuel cells. *J. Mater. Chem. A,* **3**, 14389-14400, doi:10.1039/c5ta01936b (2015).

29 Lee, K.-S. *et al.* An operationally flexible fuel cell based on quaternary ammonium-biphosphate ion pairs. *Nat. Energy,* **1**, 16120–16126, doi:10.1038/nenergy.2016.120 (2016).

30 Lee, K.-S. *et al.* Intermediate temperature fuel cells via an ion-pair coordinated polymer electrolyte. *Energy Environ. Sci.,* **11**, 979-987, doi:10.1039/c7ee03595k (2018).

31 Atanasov, V. *et al.* Synergistically integrated phosphonated polyp(pentafluorostyrene) for fuel cells. *Nat. Mater.*, published, doi:10.1038/s41563-020-00841-z (2020).

32 Venugopalan, G. *et al.* Stable and highly conductive polycation−polybenzimidazole membrane blends for intermediate temperature polymer electrolyte membrane fuel cells. *ACS Appl. Energy Mater.,* **3**, 573−585, doi:10.1021/acsaem.9b01802 (2020).

33 Bai, H. *et al.* Poly(arylene piperidine)s with phosphoric acid doping as high temperature polymer electrolyte membrane for durable, high-performance fuel cells. *J. Power Sources,* **443**, 227219|227211–227219, doi:10.1016/j.jpowsour.2019.227219 (2019).

34 Cheng, Y. *et al.* High CO tolerance of new SiO_2 doped phosphoric acid/polybenzimidazole polymer electrolyte membrane fuel cells at high temperatures of 200–250 °C. *Int. J. Hydrog. Energy,* **43**, 22487-22499, doi:10.1016/j.ijhydene.2018.10.036 (2018).

35 Zhao, W. *et al.* Self-crosslinked polyethyleneimine-polysulfone membrane for high temperature proton exchange membrane. *Acta Chimica Sinica,* **78**, 69, doi:10.6023/a19090329 (2020).

36 Tang, H. *et al.* Properties and stability of quaternary ammonium-biphosphate ion-pair poly(sulfone)s high temperature proton exchange membranes for H2/O2 fuel cells. *J. Power Sources,* **475**, 228521, doi:10.1016/j.jpowsour.2020.228521 (2020).

37 Tang, H. *et al.* Synthesis and properties of phosphonated polysulfones for durable high-temperature proton exchange membranes fuel cell. *J. Membr. Sci.,* **605**, 118107, doi:10.1016/j.memsci.2020.118107 (2020).

38 Chuang, S. *et al.* Synthesis and properties of a new fluorine-containing polybenzimidazole for high-temperature fuel-cell applications. *J. Polym. Sci., Part A: Polym. Chem.,* **44**, 4508–4513, doi:10.1002/pola.21555 (2005).

39 Qian, G. *et al.* Synthesis and characterization of high molecular weight hexafluoroisopropylidene-containing polybenzimidazole for high-temperature polymer electrolyte membrane fuel cells. *J. Polym. Sci., Part A: Polym. Chem.,* **47**, 4064-4073, doi:10.1002/pola (2009).

40 Qian, G. *et al.* Synthesis and characterization of high molecular weight perfluorocyclobutyl-containing polybenzimidazoles (PFCB–PBI) for high temperature polymer electrolyte membrane fuel cells. *Polymer,* **50**, 3911-3916, doi:10.1016/j.polymer.2009.06.024 (2009).

41 Yu, S. *et al.* Synthesis and properties of functionalized polybenzimidazoles for high-temperature PEMFCs. *Macromolecules,* **42**, 8640-8648, doi:10.1021/ma9015664 (2009).

42 Li, Q. F. *et al.* Properties, degradation and high temperature fuel cell test of different types of PBI and PBI blend membranes. *J. Membr. Sci.,* **347**, 260-270, doi:10.1016/j.memsci.2009.10.032 (2010).

43 Xu, N. *et al.* Synthesis of novel polybenzimidazoles with pendant amino groups and the formation of their crosslinked

membranes for medium temperature fuel cell applications. *J. Poly. Sci.: Part A: Pol. Chem.,* **47**, 6992-7002, doi:10.1002/pola.23738 (2009).
44 Bhadra, S. *et al.* A new self-cross-linked, net-structured, proton conducting polymer membrane for high temperature proton exchange membrane fuel cells. *J. Membr. Sci.,* **349**, 304-311, doi:10.1016/j.memsci.2009.11.061 (2010).
45 Xu, H. *et al.* Synthesis of hyperbranched polybenzimidazoles and their membrane formation. *J. Membr. Sci.,* **288**, 255-260, doi:10.1016/j.memsci.2006.11.022 (2007).
46 Bhadra, S. *et al.* Hyperbranched poly(benzimidazole-co-benzene) with honeycomb structure as a membrane for high-temperature proton-exchange membrane fuel cells. *Journal of Power Sources* **195**, 2470-2477, doi:10.1016/j.jpowsour.2009.11.083 (2010).
47 Jheng, L.-C. *et al.* A novel asymmetric polybenzimidazole membrane for high temperature proton exchange membrane fuel cells. *J. Mater. Chem. A,* **2**, 4225, doi:10.1039/c3ta14631f (2014).
48 Wang, L. *et al.* Synthesis and preparation of branched block polybenzimidazole membranes with high proton conductivity and single-cell performance for use in high temperature proton exchange membrane fuel cells. *J. Membr. Sci.,* **602**, 117981, doi:10.1016/j.memsci.2020.117981 (2020).
49 Berber, M. R. *et al.* Bipyridine-based polybenzimidazole membranes with outstanding hydrogen fuel cell performance at high temperature and non-humidifying conditions. *J. Membr. Sci.,* **591**, 117354, doi:10.1016/j.memsci.2019.117354 (2019).
50 Wang, P. *et al.* Toward enhanced conductivity of high-temperature proton exchange membranes: development of novel PIM-1 reinforced PBI alloy membranes. *Chem. Commun.,* **55**, 6491-6494, doi:10.1039/c9cc02102g (2019).
51 Li, X. *et al.* Highly conductive and Mmechanically stable imidazole-rich cross-linked networks for high-temperature proton exchange membrane fuel cells. *ACS Appl. Mater. Interfaces,* **32**, 1182-1191, doi:10.1021/acs.chemmater.9b04321 (2020).
52 Li, X. *et al.* Construction of high-performance, high-temperature proton exchange membranes through incorporating SiO_2 nanoparticles into novel nross-linked polybenzimidazole networks. *ACS Appl. Mater. Interfaces,* **11**, 30735-30746, doi:10.1021/acsami.9b06808 (2019).
53 Li, X. *et al.* Arylether-type polybenzimidazoles bearing benzimidazolyl pendants for high-temperature proton exchange membrane fuel cells. *J. Power Sources,* **393**, 99-107, doi:10.1016/j.jpowsour.2018.05.011 (2018).
54 Li, X. *et al.* Dimensionally-stable phosphoric acid–doped polybenzimidazoles for high-temperature proton exchange membrane fuel cells. *J. Power Sources,* **336**, 391-400, doi:10.1016/j.jpowsour.2016.11.013 (2016).
55 Jang, J. *et al.* Phosphoric acid doped triazole-containing cross-linked polymer electrolytes with enhanced stability for high-temperature proton exchange membrane fuel cells. *J. Membr. Sci.* **595**, 117508, doi:10.1016/j.memsci.2019.117508 (2020).
56 Cao, Y.-C. *et al.* A polytetrafluoroethylene porous membrane and dimethylhexadecylamine quaternized poly (vinyl benzyl chloride) composite membrane for intermediate temperature fuel cells. *J. Power Sources,* **294**, 691-695, doi:10.1016/j.jpowsour.2015.06.113 (2015).
57 Zhang, S. *et al.* Unusual influence of binder composition and phosphoric acid leaching on oxygen mass transport in catalyst layers of high-temperature proton exchange membrane fuel cells. *J, Power Sources,* **473**, 228616, doi:10.1016/j.jpowsour.2020.228616 (2020).
58 Xu, X. *et al.* A novel phosphoric acid doped poly(ethersulphone)-poly(vinyl pyrrolidone) blend membrane for high-temperature proton exchange membrane fuel cells. *J.Power Sources,* **286**, 458-463, doi:10.1016/j.jpowsour.2015.04.028 (2015).
59 Guo, Z. *et al.* New anhydrous proton exchange membranes for high-temperature fuel cells based on PVDF–PVP blended polymers. *J. Chem. Mater. A,* **3**, 148-155, doi:10.1039/c4ta04952g (2015).
60 Pan, C. *et al.* Preparation and operation of gas diffusion electrodes for high-temperature proton exchange membrane fuel cells. *J. Power Sources,* **172**, 278-286, doi:10.1016/j.jpowsour.2007.07.019 (2007).
61 Tang, H. *et al.* Fuel cells with an operational range of -20 to 200 °C enabled by phosphoric acid-doped intrinsically ultramicroporous membranes. *Nat. Energy,* **7**, 153–162, doi: 10.1038/s41560-021-00956-w (2022).

62 Lim, K. H. *et al.* Protonated phosphonic acid electrodes for high power heavy-duty vehicle fuel cells. *Nat. Energy*, doi:10.1038/s41560-021-00971-x (2022).

63 Liang, H. *et al.* Development of membrane electrode assembly for high temperature proton exchange membrane fuel cell by catalyst coating membrane method. *J. Power Sources,* **288**, 121-127, doi:10.1016/j.jpowsour.2015.04.123 (2015).

64 Liang, H. *et al.* Membrane electrode assembly with enhanced platinum utilization for high temperature proton exchange membrane fuel cell prepared by catalyst coating membrane method. *J. Power Sources,* **266**, 107-113, doi:10.1016/j.jpowsour.2014.05.014 (2014).

65 Yao, D. *et al.* Achieving high Pt utilization and superior performance of high temperature polymer electrolyte membrane fuel cell by employing low-Pt-content catalyst and microporous layer free electrode design. *J. Power Sources,* **426**, 124-133, doi:10.1016/j.jpowsour.2019.04.045 (2019).

66 Úbeda, D. *et al.* Life test of a high temperature PEM fuel cell prepared by electrospray. *Int. J. Hydrog. Energy,* **41**, 20294-20304, doi:10.1016/j.ijhydene.2016.09.109 (2016).

67 Liu, F. *et al.* Influence of the interaction between phosphoric acid and catalyst layers on the properties of HT-PEFCs. *Fuel Cells,* **14**, 750-757, doi:https://doi.org/10.1002/fuce.201300272 (2014).

68 Su, H. *et al.* Low platinum loading for high temperature proton exchange membrane fuel cell developed by ultrasonic spray coating technique. *J. Power Sources,* **267**, 155-159, doi:10.1016/j.jpowsour.2014.05.086 (2014).

69 Martin, S. *et al.* Binderless electrodes for high-temperature polymer electrolyte membrane fuel cells. *J. Power Sources,* **272**, 559-566, doi:10.1016/j.jpowsour.2014.08.112 (2014).

70 Martin, S. *et al.* Lowering the platinum loading of high temperature polymer electrolyte membrane fuel cells with acid doped polybenzimidazole membranes. *J. Power Sources,* **293**, 51-56, doi:10.1016/j.jpowsour.2015.05.031 (2015).

71 Martin, S. *et al.* Feasibility of ultra-low Pt loading electrodes for high temperature proton exchange membrane fuel cells based in phosphoric acid-doped membrane. *Int. J. Hydrog. Energy,* **44**, 28273-28282, doi:10.1016/j.ijhydene.2019.09.073 (2019).

72 Martin, S. *et al.* Ten-fold reduction from the state-of-the-art platinum loading of electrodes prepared by electrospraying for high temperature proton exchange membrane fuel cells. *Electrochem. Commun.,* **93**, 57-61, doi:10.1016/j.elecom.2018.06.007 (2018).

73 Kim, S. *et al.* The effect of binder content on the performance of a high temperature polymer electrolyte membrane fuel cell produced with reactive spray deposition technology. *Electrochim. Acta,* **177**, 190-200, doi:10.1016/j.electacta.2015.02.025 (2015).
