# Supplementary material for: Low Pt loading for high-performance fuel cell electrodes enabled by hydrogen-bonding microporous polymer binders
Source: Nat Commun. 2022 Dec 8;13:7577. doi: 10.1038/s41467-022-34489-x (PMC9732346; doi:10.1038/s41467-022-34489-x)
Supplement: Supplementary file 3 — Description to Additional Supplementary Information [file 41467_2022_34489_MOESM3_ESM.pdf]

Description of Additional Supplementary Files

Supplementary Dataset file 1: The coordinates for the calculation of PA interaction with binder materials.

Supplementary Dataset file 2: The coordinates for the calculation of binder materials' pKa value.
